# Persistent Homology-Based Machine Learning Method for Filtering and Classifying Mammographic Microcalcification Images in Early Cancer Detection

**DOI:** 10.3390/cancers15092606

**Published:** 2023-05-04

**Authors:** Aminah Abdul Malek, Mohd Almie Alias, Fatimah Abdul Razak, Mohd Salmi Md Noorani, Rozi Mahmud, Nur Fariha Syaqina Zulkepli

**Affiliations:** 1 Department of Mathematical Sciences, Faculty of Science & Technology, Universiti Kebangsaan Malaysia (UKM), Bangi 43600, Selangor, Malaysia; 2 Mathematical Sciences Studies, College of Computing, Informatics and Media, Universiti Teknologi MARA (UiTM) Negeri Sembilan Branch, Seremban Campus, Seremban 70300, Negeri Sembilan, Malaysia; 3 Centre for Modelling and Data Analysis (DELTA), Faculty of Science & Technology, Universiti Kebangsaan Malaysia (UKM), Bangi 43600, Selangor, Malaysia; 4 Department of Radiology and Imaging, Faculty of Medicine and Health Sciences, Universiti Putra Malaysia (UPM), Serdang 43400, Selangor, Malaysia; 5 School of Mathematical Sciences, Universiti Sains Malaysia (USM), Gelugor 11800, Penang, Malaysia

**Keywords:** topological data analysis, persistent homology, microcalcification, filtering, image processing

## Abstract

**Simple Summary:**

The appearance of microcalcifications in mammogram images is an essential predictor for radiologists to detect early-stage breast cancer. This study aims to demonstrate the strength of persistent homology (PH) in noise filtering and feature extraction integrated with machine learning models in classifying microcalcifications into benign and malignant cases. The methods are implemented on two public mammography datasets: the Mammographic Image Analysis Society (MIAS) and Digital Database for Screening Mammography (DDSM). This study discovered that PH-based machine learning techniques can improve classification accuracy, which could benefit radiologists and clinicians in early diagnosis.

**Abstract:**

Microcalcifications in mammogram images are primary indicators for detecting the early stages of breast cancer. However, dense tissues and noise in the images make it challenging to classify the microcalcifications. Currently, preprocessing procedures such as noise removal techniques are applied directly on the images, which may produce a blurry effect and loss of image details. Further, most of the features used in classification models focus on local information of the images and are often burdened with details, resulting in data complexity. This research proposed a filtering and feature extraction technique using persistent homology (PH), a powerful mathematical tool used to study the structure of complex datasets and patterns. The filtering process is not performed directly on the image matrix but through the diagrams arising from PH. These diagrams will enable us to distinguish prominent characteristics of the image from noise. The filtered diagrams are then vectorised using PH features. Supervised machine learning models are trained on the MIAS and DDSM datasets to evaluate the extracted features’ efficacy in discriminating between benign and malignant classes and to obtain the optimal filtering level. This study reveals that appropriate PH filtering levels and features can improve classification accuracy in early cancer detection.

## 1. Introduction

Female breast cancers continue to record increases in the numbers of new cases and are reported as the most common incidence and mortality cancer worldwide, surpassing other types of cancer [1]. According to the World Health Organization (WHO), more than 2.3 million women were diagnosed with breast cancer globally in 2020, and 685,000 died from the disease [2]. This means that, on average, a woman is diagnosed with breast cancer every 14 s. Preventive measures, including imaging screening, can potentially detect cancer at an early stage, which in turn increases the patient’s survival rate [3]. Among the numerous screening methods currently available for the detection of breast cancer, mammography has become the main and most effective imaging method. Apart from that, it has increased the rate of early-stage cancer detection [4,5].

**Figure 1:**
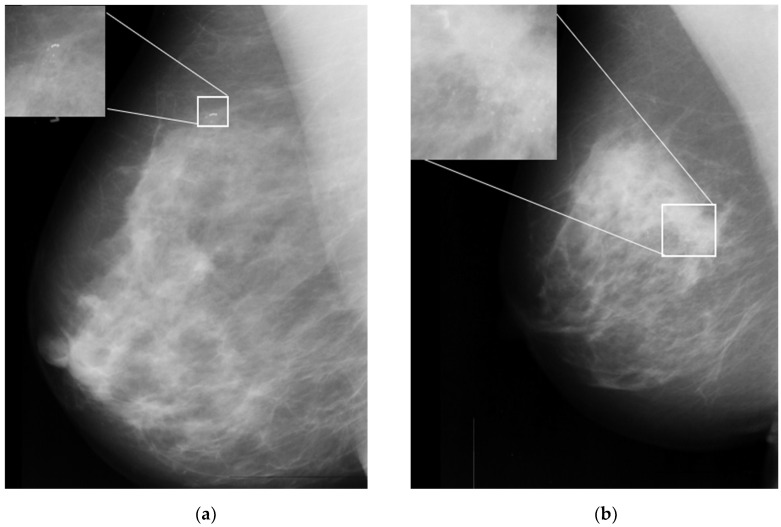
Sample microcalcification on mammogram images in the MIAS dataset [7]. (**a**) Benign microcalcifications and (**b**) malignant microcalcifications.

One of the important signs in cancer detection on mammographic images is the presence of microcalcifications, which appear as small bright spots within an inhomogeneous background [6]. Figure 1a,b illustrates an example of benign and malignant microcalcifications on mammogram images taken from the MIAS database [7]. Note that the morphology of this microcalcification is a crucial predictor of its pathological nature. Large, round, and oval calcifications of uniform size exhibit benign (non-cancerous) characteristics. In contrast, smaller and non-uniform calcifications exhibit characteristics of malignant growth [8,9]. In clinical practice, it is difficult and time-consuming for radiologists to interpret and evaluate microcalcifications accurately. This is true especially when the microcalcifications appear in low contrast and are obscured by the background tissue of the images [10]. Here, human errors based on subjective evaluations may lead to unnecessary biopsy procedures, which can cause harm and anxiety for patients [11].

**Figure 2:**
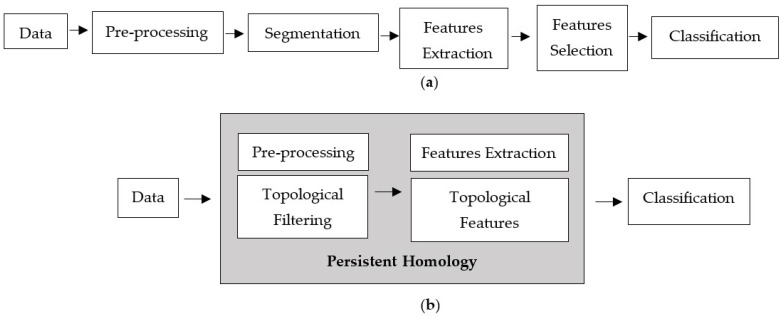
Computer-aided detection processes. (**a**) Standard processes and (**b**) proposed techniques.

In order to improve the accuracy of assessing microcalcifications, numerous studies have been conducted to develop computational approaches that could potentially aid radiologists in distinguishing benign and malignant microcalcifications [3]. The standard computer-aided detection (CAD) processes consist of image preprocessing, segmentation, feature extraction, feature selection, and classification model, as depicted in Figure 2a. Each phase involves a different technique, and the performance relies heavily on the preceding phase. Preprocessing is the initial phase of the image processing pipeline. Filtering is commonly applied as a preprocessing technique for removing noises and other artefacts in the image. Noise emerges in mammograms when the image’s brightness varies in areas representing the same tissues owing to non-uniform photon distribution [3]. It produces a grainy appearance, reducing the visibility of some features within the image, especially microcalcifications in dense breast tissue. Because noise, edge, and texture are high-frequency components, distinguishing them is challenging [12].

Various filtering techniques have been used in the literature to reduce noise in mammogram images. Each method has its benefits and drawbacks. For example, the Wiener filter is considered a linear filter that can improve the images by reducing random noise but may produce a blurry effect and incomplete noise filtration [13]. Non-linear filters, such as the median filter, can overcome the limitations of linear filters thanks to several benefits, such as being straightforward and offering a sensible noise removal performance, but they could distort fine edges even at low noise densities [14]. The comparative reviews from [12] agreed that different types of noise require different filtering techniques.

Another important phase that influences classification performance is feature extraction [11]. Feature extraction methodologies analyse images to extract the most prevalent features and are employed as inputs to machine learning classifiers to distinguish between benign and malignant classes. Such features included intensity, statistical, shape, and textural features [5]. The grey-level cooccurrence matrix (GLCM), which calculates the occurrence of various grey levels in a region of interest (ROI), is a well-known texture feature and is utilised extensively in the literature [5,15,16,17]. Nevertheless, all of these features focus on local information of the images and are often burdened with details, resulting in data complexity [18].

This study proposes a new classification approach based on persistent homology (PH) that can extract informative features from the images, which consists of filtering and feature extraction processes, as shown in Figure 2b.

PH, the topological data analysis (TDA) core tool, has recently been widely used as a multi-scale representation of topological features. It can extract topological summaries from data that capture the birth and death of connected components, loops, and voids through a filtration process [19]. Apart from that, persistence diagrams (PD) are one of the topological descriptors produced by PH [20]. They comprise a collection of points in the half-plane above the diagonal with coordinates (birth and death) of topological features, helpful in distinguishing robust and noisy topological properties [21]. Other than that, the geometric measurement of the associated topological properties directly correlates with the lifespan (differences between death and birth). A long lifespan is considered a prominent feature represented by points far from the diagonal in the diagram. In contrast, short lifespans, represented by points close to the diagonal, are interpreted as noise [22].

There has been minimal effort to explore the potential use of PD as a filtering approach, especially in mammogram images. A noteworthy study by [23] filtered out 20% of points close to the diagonal in PD, but they focused on interpreting quality assessment in the eye fundus image. The PD of a microcalcification image contains thousands of points with many short lifespans (noise), necessitating an additional filtering procedure. Thus, a novel method for filtering noise in a persistent diagram based on the maximum lifespan of the image is proposed.

In PH, the topological features generated from PD can be vectorised and integrated into machine learning models. Various vectorisation approaches have been proposed, with promising results in various fields. For example, the persistent image (PI) feature proposed by [24] has been utilised in hepatic tumour classification with considerable accuracy [25,26]. Meanwhile, the persistent entropy (PE) and p-norm features were applied for dark soliton detection [27] and persistent landscapes (PL) in the quantitative analysis of fluorescence microscopy images [28]. Furthermore, the authors of [29] employed Betti numbers for evaluating tumour heterogeneity in image feature extraction. Nevertheless, a recent study by [30] stated that keeping track of the lifespan is more informative than the progression of Betti numbers. The researchers used the mean, the standard deviation of lifespan for each cycle, and PE for 0- and 1-dimensional features to embed in machine learning techniques in detecting the correct Gleason score of prostate cancer, reporting an accuracy above 95%.

PH-based machine learning is a promising technique with many potential applications across different fields [31]. However, one of the challenges mentioned in the published literature is PH-based feature representation [19]. In other words, selecting suitable topological features is crucial because the suitability of features depends on the data type and the problem at hand. This study explored the potential use of the PH method for noise filtering and feature extraction processes. To the best of our knowledge, this is the first work using PH to tackle the challenge of filtering noise and selecting suitable topological features to improve the classification performance of microcalcifications in mammogram images. The purpose of this paper can be summarised as follows: (i) to propose multi-level noise filtering of 1-dimensional homology group PD based on maximum lifespan, (ii) to obtain the vectorised topological features from the filtered PD using the PI and PE, (iii) to compare the performance of the filter and non-filter PD including the performance of an individual feature and concatenated features using several machine learning models, as well (iv) to suggest the optimal filtration level for the MIAS and DDSM datasets. As this work aims to highlight the importance of a topological approach to classify the microcalcifications in a machine learning setting, prior knowledge of machine learning is assumed. Therefore, it will not be recalled in this section or elsewhere in the paper.

## 2. Materials and Methods

**Figure 3:**
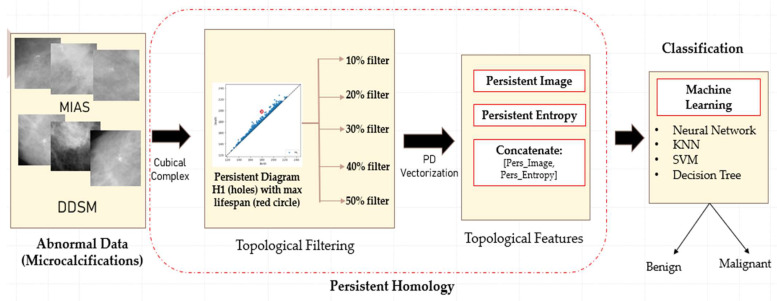
Illustration of the proposed classification framework using persistent homology.

The proposed classification framework consists of four main steps: data acquisition, topological filtering, topological features vectorisation, and classification using machine learning classifiers, as illustrated in Figure 3.

### 2.1. Dataset

The data utilised in this study consist of two publicly available datasets. The first dataset was taken from the MIAS dataset [7], containing 26 image patches with a size of 200 × 200 pixels. These patches were cropped manually as the centre and radius of microcalcification clusters are provided. There were thirteen malignant and benign cases, each with three types of background tissue: fatty, fatty glandular, and dense glandular. The second dataset was taken from the digital database for screening mammography (DDSM) dataset [32], consisting of 140 image patches randomly selected with seventy cases for each of malignant and benign cases. The size of the images is set to 300 × 300 pixels. Subsequently, the diagnostics status of each patch is either benign or malignant, annotated by radiologists based on biopsy results. Despite the large number of mammograms in these two datasets, not all have microcalcifications. Therefore, the number of microcalcification patches is substantially lower than the total number of images in the datasets.

### 2.2. Persistent Homology

PH is a primary technique in TDA based on the concept that topological features detected over varying scales are more likely to represent intrinsic features [33]. Other than that, PH studies geometric patterns by looking at the evolution of k-dimensional holes, such as 0-dimensional holes (H_0_) representing connected components, 1-dimensional holes (H_1_) representing loops, and 2-dimensional holes (H_2_) representing voids.

**Figure 4:**
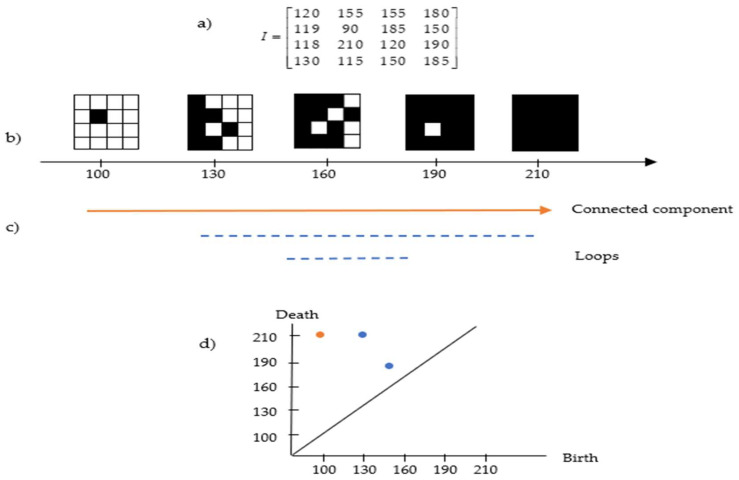
Example of the cubical complex in a greyscale image. (**a**) Image pixel values; (**b**) the filtered cubical complex; (**c**) the lifespan of connected components and loops based on (**b**); and (**d**) the persistent diagram based on (**c**).

Images can be interpreted as geometric shapes known as cubical complexes. In 2D greyscale images, the cubical complexes are topological spaces consisting of a combination of 0-cube (vertices), 1-cube (edges), and 2-cube (squares). It can be efficiently constructed by assigning a vertex to each pixel, then combining vertices corresponding to nearby pixels by an edge and filling process in the resulting squares [34]. Let I be a greyscale image of size N×M with grey intensities $2^{n}$ (we use an 8-bit greyscale n=8). The pixel intensity value of I on the intervals $\left[0,2^{n}-1\right]$ is [0,255], where 0 represents black colour and 255 represents white colour, with shades of grey for the values in between. Once these images are interpreted as 2-cubical simplices, greyscale filtration with 256 sublevels can be constructed as a nested sequence $K_{0}\subset K_{1}\subset K_{2}\subset \ldots \subset K_{2^{n}-1}$ called sublevel set filtration [25] (see the example in Figure 4).

Figure 4a,b present an example of cubical complex filtration in a matrix representing the pixel value of the image. The lifespan can be seen in Figure 4c, where the connected component appears at the intensity of 100 and continues to exist because all subsequent pixels are always connected to the preceding ones. At filtration 120, a loop exists (full white pixels inside components made of black pixels) and deaths exist at filtration 210. Apart from that, another loop exists at filtration 150 and deaths shortly after at filtration 185. This information can be transformed in the persistent diagram representation as in Figure 4d, which will be discussed in the following section.

#### 2.2.1. Interpreting the Persistent Diagrams

**Figure 5:**
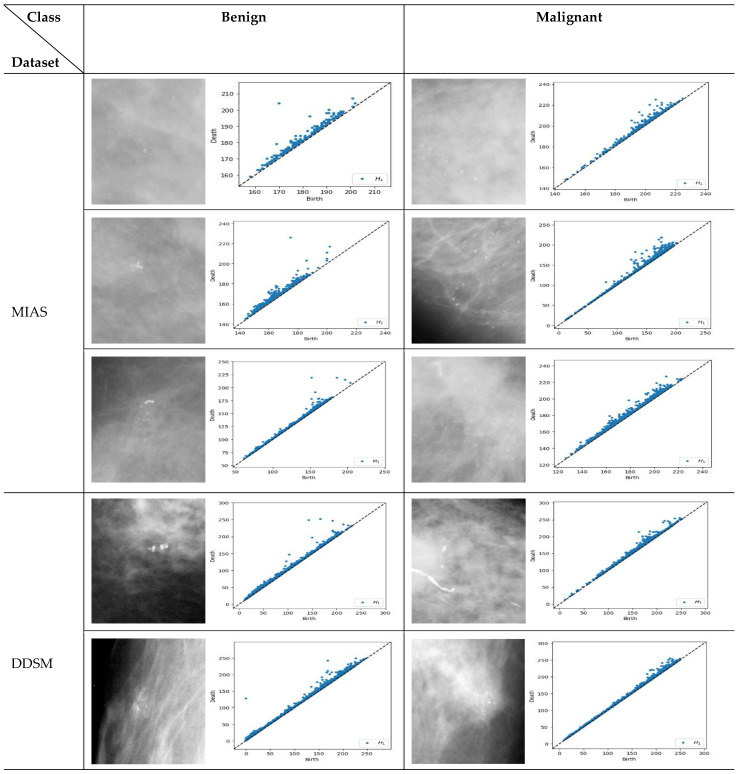
Persistent diagram of H_1_ (loops) for benign and malignant microcalcifications in the MIAS [7] and DDSM datasets [32]. For the MIAS dataset, the first row is a sample of dense glandular tissue, the second is a sample of fatty tissue, and the third is fatty glandular tissue.

PD represents a pattern-generating field and provides a (visual) summary of the set of points $\left\{(b,d)|b,d\in {\mathbb{R}}^{2}\right\}$ and *d > b*, where the births (*b*) are marked on the x-axis and deaths (*d*) on the y-axis. The lifespan *d-b >* 0 indicates the prominence of features. Long-lived features are those far from the diagonal line. They are usually regarded as significant features and represent the robustness of holes against noise, whereas short lifespans are represented by points close to the diagonal and are interpreted as noise [25,35]. All points of the different homological dimensions are present in PD, such as 0-dimensional holes denoted as H_0_ (connected complete black pixels) and 1-dimensional holes denoted as H_1_ or loops (complete white pixels inside components made of black pixels). However, some dimensions may be irrelevant to the study [31]. As microcalcifications appear as white spots, this study employs only H_1_. Figure 5 illustrates samples of PD for H_1_ obtained from benign and malignant microcalcifications in different datasets.

Similar structure of the images will have comparable pattern in PDs [36]. For example, based on Figure 5, the benign microcalcifications indicate a similar pattern for different types of density and datasets where topological features associated with long-lived lifespan are observed. Conversely, in malignant microcalcifications, the PD illustrates short-lived features where the points are tightly packed and close to the diagonal. Furthermore, it is known that the points close to the diagonal line are usually regarded as less “useful” and linked with noise [37,38]. Thus, multi-level filtering of PDs offers a rigorous solution to the problem of distinguishing between anomalies and noise in these representations. The proposed procedures are described in the following steps:

Step 1:  Obtain PDs for 1-dimensional holes (H_1_);
Step 2:  Calculate the lifespan for each point in the PD;
Step 3:  Find the maximum lifespan;
Step 4:  Filter.

$$\begin{array}{c} \mathrm{Filter}\_10\%=\mathrm{lifespan}>(0.1*\max\_\mathrm{lifespan})\\ \mathrm{Filter}\_20\%=\mathrm{lifespan}>(0.2*\max\_\mathrm{lifespan})\\ \mathrm{Filter}\_30\%=\mathrm{lifespan}>(0.3*\max\_\mathrm{lifespan})\\ \mathrm{Filter}\_40\%=\mathrm{lifespan}>(0.4*\max\_\mathrm{lifespan})\\ \mathrm{Filter}\_50\%=\mathrm{lifespan}>(0.5*\max\_\mathrm{lifespan}) \end{array}$$

**Figure 6:**
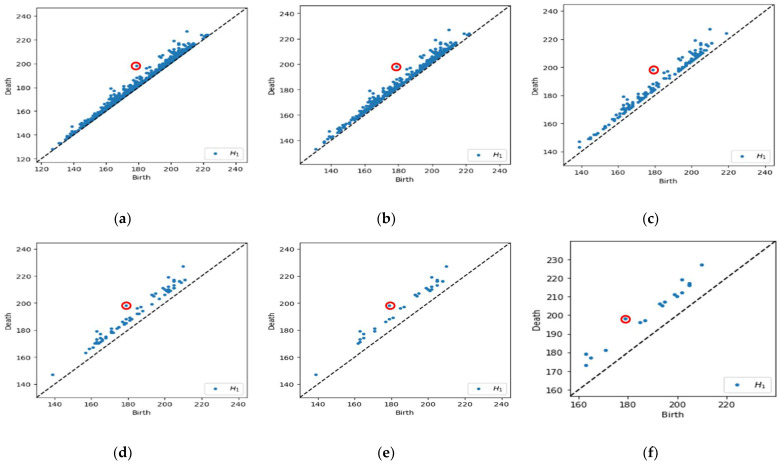
Example of a multi-level filtering PD with Betti number (B_1_). (**a**) Original PD with max lifespan (red circle), B_1_ = 1341; (**b**) 10% of data filtered out, B_1_ = 510; (**c**) 20% of data filtered out, B_1_ = 162; (**d**) 30% of data filtered out, B_1_ = 68; (**e**) 40% of data filtered out, B_1_ = 32; and (**f**) 50% of data filtered out, B_1_ = 18.

We choose the filter interval range from 10% to 50% with 10% increments to facilitate interpretation when determining which level yields the best classification results for each dataset. The number of points in the PD can be reported as Betti numbers, denoted as B_1_ for H_1_. Hence, the filtering process will reduce the number of B_1_ values. Figure 6 demonstrates a schematic representation of a multi-level filtering PD in a sample of a malignant image.

Referring to Figure 6a, we calculate the lifespan for each point in the PD and obtain the maximum lifespan. In Figure 6b–f, the points are filtered out based on a lifespan greater than 10% to 50% of the maximum lifespan, which implies the number of B_1_ reduced from 1341 points initially to 510, 162, 68, 32, and 18, respectively. Subsequently, the original and filtered PD is then used to obtain the feature vector representations, which can be easily incorporated as input in machine learning models.

#### 2.2.2. Vectorised Topological Features

Vectorising topological features in PH converts the topological features obtained from a PD into a vector representation that can be employed for machine learning models. This study employs two topological features so that the distribution of the persistence feature (lifespan) can be characterised as either benign or malignant. The vectorised topological features used are described as follows.

- Persistent Entropy (PE)

PE measures the disorder in the distribution of lifespan (persistence). Let $PD={\left\{(b_{j},d_{j})\right\}}_{j\in I}$ be a persistent diagram where $b_{j}$ and $d_{j}$ are the birth and death points, respectively, of the topological structure in pixel image *I* and lifespan 𝓁=|d−b|. The entropy of PD is defined as follows [23,39]:

(1) $$\varepsilon (PD)=\sum_{j=1}^{n}\frac{{𝓁}_{j}}{S({𝓁}_{j})}\log\left(\frac{{𝓁}_{j}}{S({𝓁}_{j})}\right)$$

where $S({𝓁}_{j})$ is the sum of the lifespan in the PD. Every PD will produce one entropy value. Based on [27], an entropy value of 0 represents a single feature in the image, while *N* features consist of ε(PD)=log(N).

- Persistent Image (PI)

PI is one of the prevalent vectorisation methods adopted to convert the topological information in PDs into a finite-dimensional vector representation. It was introduced by Adams et al. [24]. The PD, which consists of the persistence point at birth (b)/death (d) coordinates, is rotated by $\frac{\pi }{4}$ to construct the PI. Consequently, the rotated PD denoted as *R* can be discretised into the persistent surface (ρ) in (x,y) coordinates, defined as

(2) $$\rho R(x,y)=\sum_{(b,d)\in R}g_{\left(b,d\right)}(x,y)\cdot f(b,d),$$

where $g_{\left(b,d\right)}$ is a gaussian smoothing function given by

(3) $$g_{\left(b,d\right)}(x,y)=\frac{1}{2\pi \sigma^{2}}e^{-[{\left(x-b\right)}^{2}+{\left(y-d\right)}^{2}]/2\sigma^{2}},$$

and f(b,d)≥0 are non-negative weighting functions [40]. Finally, the PI can be obtained by integrating the persistence surface function ρR(x,y) over each pixel [24].

### 2.3. Machine Learning Classifier

Once the topological features are vectorised, the dataset can be implemented in the following machine learning classifiers:

- Neural network (NN);
- Support vector machine (SVM);
- K-nearest neighbour (KNN);
- Decision tree (DT).

The machine learning models are applied for comparison purposes to evaluate the performance of individual and concatenated features and to determine the optimum filtering level for each dataset.

### 2.4. Performance Evaluation

**Figure 7:**
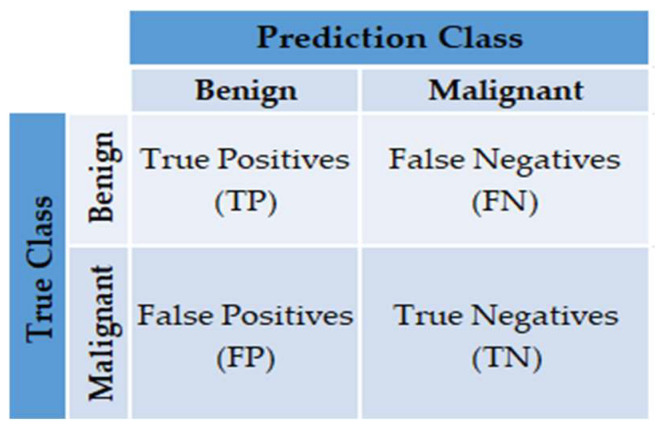
The confusion matrix.

Many different evaluation metrics can be used to measure how well the method and classifier work. This study uses the confusion matrix, accuracy, and area under the receiver operating characteristic curve (AUC). The metrics used to evaluate the results are described below [41]:

- Confusion matrix: Provide a matrix as output that describes the method’s performance consisting of the total number of correct and incorrect predictions. The matrix is shown in Figure 7.
- Classification Accuracy (CA): The percentage of microcalcifications correctly classified to the total number of observations. It can be measured as follows:
  (4) $$CA=\frac{TP+TN}{TP+TN+FP+FN}.$$
- Area under the Curve (AUC): The AUC can be measured by calculating the area under the receiver operating characteristic (ROC) curve. The ROC curve is a plot of the true positive rate (TPR), called sensitivity or recall, versus the false positive rate (FPR). TPR is defined in this context as the number of correctly diagnosed malignant cases divided by the total number of malignant cases. In contrast, FPR is defined as the number of benign cases wrongly classified as malignant divided by the total number of benign instances. TPR and FPR can be calculated using Equations (4) and (5), respectively:
  (5) $$TPR=\frac{TP}{TN+FN}.$$
  $$FPR=\frac{FP}{FP+TN}.$$

### 2.5. Implementation Details

The cubical complex filtration and H1 of PD are generated in all experiments using an open-source program, Cubical Ripser (https://github.com/shizuo-kaji/CubicalRipser_3dim/, accessed on 2 February 2022) [42]. The vectorised topological features consisting of PI and PE features can be obtained using Scikit-TDA library written in Python (https://persim.scikit-tda.org/, accessed on 19 May 2022). The PI parameters, such as pixel size, are set to 1, meaning that one vectorises the PI value for every PD. Other parameters, such as the weighting function, f(b,d), are based on the persistence values [24], and the smoothing parameter in the Gaussian function σ is set to default.

MATLAB R2021b classification learner is implemented with a fivefold cross-validation approach for machine learning models. The input data for all models are vectorised topological features, i.e., PI and PE values for each image. Each experimental test uses 11th Gen Intel(R) Core(TM) i7-11800H, CPU 2.30 GHz, 16 GB memory, and NVIDIA Geforce RTX 3050Ti for the graphics card. The hyperparameters used for each model are described as follows:

- Neural network (NN): classifier type = medium, the number of fully connected layers = 1, the first layer size = 25, and the activation function = ReLu.
- Support vector machine (SVM): kernel type = linear, kernel scale = automatic, and box constraint level = 1
- K-nearest neighbour (KNN): classifier type = fine, number of neighbours = 1, distance metric = Euclidean, and distance weight = equal.
- Decision tree (DT): classifier type = fine tree, the maximum number of splits = 100, and split criterion = Gini’s diversity index.

## 3. Results

This section presents the results based on the proposed method discussed in Section 2 applied to two public datasets, MIAS and DDSM. Furthermore, this section discusses the usefulness of the PH approach in discriminating the topological features between two classes, benign and malignant. The performances of single and concatenate features are also examined using four machine learning classifiers by comparing the CA and AUC. Finally, the optimal topological filter for each dataset is also presented.

### 3.1. Topological Filtering

**Figure 8:**
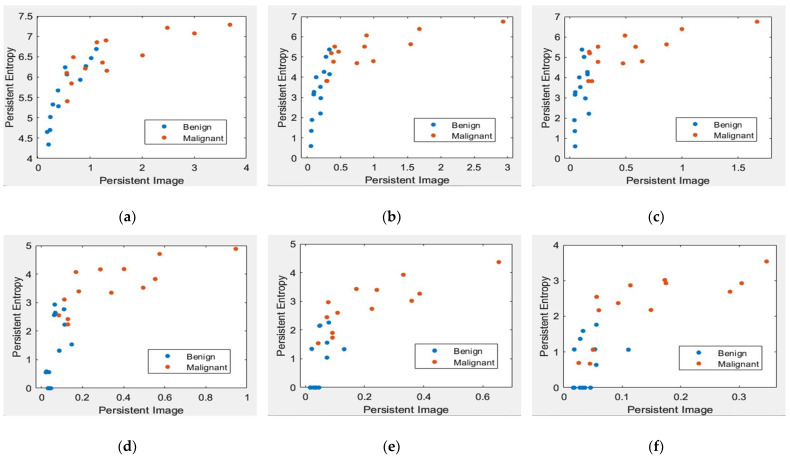
Scatter plot of PE versus a PI with different levels of filters in the MIAS dataset [7]. (**a**) Non-filter; (**b**) filter 10% of max lifespan; (**c**) filter 20% of max lifespan; (**d**) filter 30% of max lifespan; (**e**) filter 40% of max lifespan; and (**f**) filter 50% of max lifespan.

**Figure 9:**
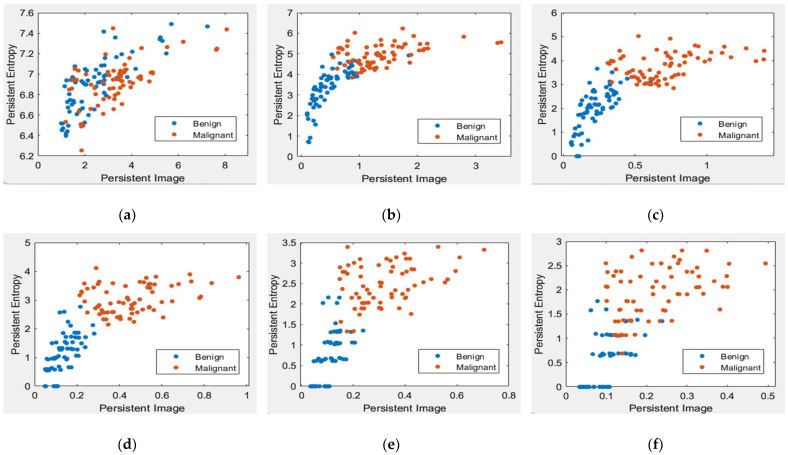
Scatter plot of persistent entropy versus a persistent image with different levels of filters in the DDSM dataset [32]. (**a**) Non-filter; (**b**) filter 10% of max lifespan; (**c**) filter 20% of max lifespan; (**d**) filter 30% of max lifespan; (**e**) filter 40% of max lifespan; and (**f**) filter 50% of max lifespan.

Figure 8 and Figure 9 illustrate the scatter plot of PE versus PI feature values with different levels of topological filtering in the MIAS and DDSM datasets, respectively.

Figure 8 and Figure 9 demonstrate scatter plots where each point corresponds to the value of PE versus PI. The number of points represents 26 images in the MIAS dataset and 140 in the DDSM dataset. Note that non-filtered PD in Figure 8a and Figure 9a refers to the original images containing noise. Noise can cause spurious features to appear in the image, leading to incorrect topological inferences. This is because noise can introduce false persistence of features that are not truly persistent and may also obscure the persistence of real features. Thus, the separation between two classes, benign and malignant, is more difficult to distinguish by the value of PE and PI features. Removing the short lifespan or noise through PD filtration at 10 to 30% provides a positive impact, where the benign class tends to have smaller values of PE and PI than the malignant class, as in Figure 8 and Figure 9b–d. However, high PD filters (40% and 50%) cause the PE and PI values to overlap, as in Figure 8 and Figure 9e–f.

### 3.2. Classification Performance

The optimum filtering level was selected by comparing the individual’s performance and concatenating topological features using four machine learning classifiers, namely, the NN, KNN, SVM, and DT models. Table 1 and Table 2 present the performance of the persistence image feature, while Table 3 and Table 4 present the PE feature in the MIAS and DDSM datasets, respectively.

It can be observed that the classification performance from all classifiers is improved when the filtering process is applied. Table 1 and Table 3 present that the optimal filtering level for persistent images and entropy features in the MIAS dataset is at a filter of 20%. Here, NN, DT, and KNN attain an accuracy of 92.3% for both topological features with an AUC value of 0.9 and above. For the DDSM dataset in Table 2 and Table 4, filter 30% delivered remarkable results, with all classifiers achieving above 92.9% accuracy and up to 0.99 AUC. Note that the performance in the DDSM dataset is more consistent in all classifiers because it utilised more images (140 images) than the MIAS dataset, which only used 26 images.

The classification performance can be significantly improved by concatenating features (multi-vector). In the MIAS dataset, the performance is improved from 92.3% to 96.2% accuracy and, in the DDSM dataset, it is increased to 99.3%, as shown in Table 5 and Table 6.

### 3.3. Performance of Machine Learning Models

**Figure 10:**
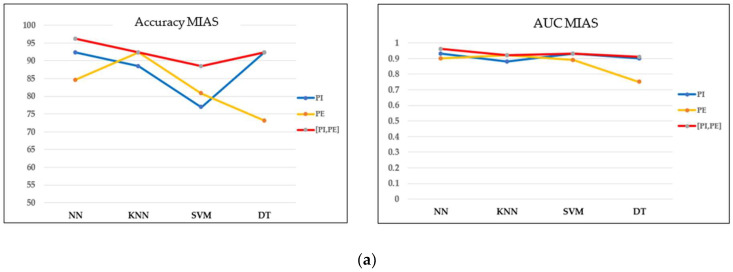

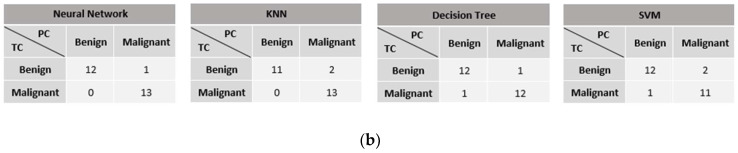
Performance comparison of machine learning classifiers based on the optimal filter (20%) in the MIAS dataset. TC = true class and PC = predicted class. (**a**) Accuracy and AUC of classifiers. (**b**) Confusion matrix of concatenating features.

Based on the optimal filtering level in both datasets, the performances of four classifiers were evaluated and compared in terms of accuracy, the AUC, and the confusion matrix. Referring to the MIAS dataset in Figure 10a,b, most models perform well on concatenating features with greater than 0.9 AUC and a maximum of three misclassified cases in the confusion matrix. Moreover, the accuracy obtained is greater than 90%, except for the SVM model. As this dataset contains three different types of densities, particularly when the breast density is high, the microcalcifications could be obscured by the dense tissues, making it more challenging to classify them. These findings align with the results reported by [43], where the SVM accuracy drops because of the greyscale image’s brightness. On the other hand, NN indicated the best performance model for this dataset, with the highest accuracy and AUC and only one false negative (FN) case in the confusion matrix.

**Figure 11:**
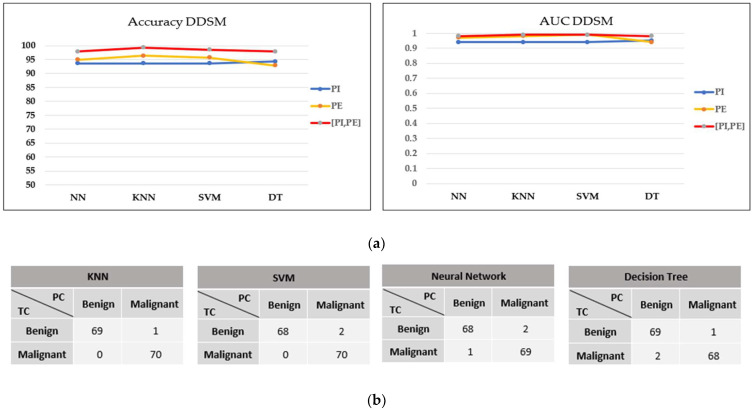
Performance comparison of machine learning classifiers based on the optimal filter (30%) in the DDSM dataset. (**a**) Accuracy and AUC of classifiers. (**b**) Confusion matrix of concatenating features.

In contrast, all models in the DDSM dataset exhibit outstanding classification performance on concatenating features, with greater than 95% accuracy, greater than 0.97 AUC, and a maximum of three misclassified cases in the confusion matrix, as shown in Figure 11a,b. The KNN model performed the best in this dataset, with the highest accuracy and only one FN result.

## 4. Discussion and Future Work

The literature commonly describes PHs robust against image noise [25,31]. This study demonstrates that if the PD is taken directly without any filtering on the diagram, machine learning models cannot successfully classify the vectorised topological features of microcalcification. Other than that, experimental results indicate that the performance was improved by implementing the optimal filtering level for each dataset.

**Figure 12:**
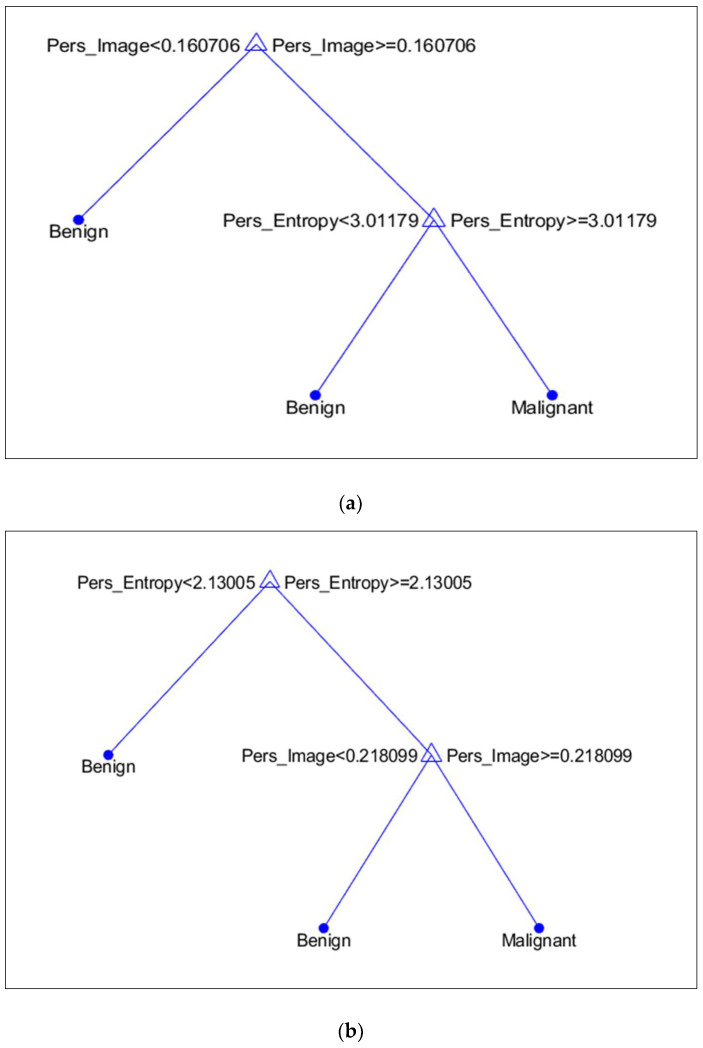
Discriminant feature values based on the Decision Tree model. (**a**) MIAS Dataset; (**b**) DDSM Dataset.

It is discovered that, in terms of topological features, PI is more prominent in the MIAS dataset, whereas PE in the DDSM dataset. Figure 12a,b illustrates the discriminant values of every feature based on the DT model. Compared to malignant microcalcifications, benign microcalcifications will have a lower value of PI and PE.

**Figure 13:**
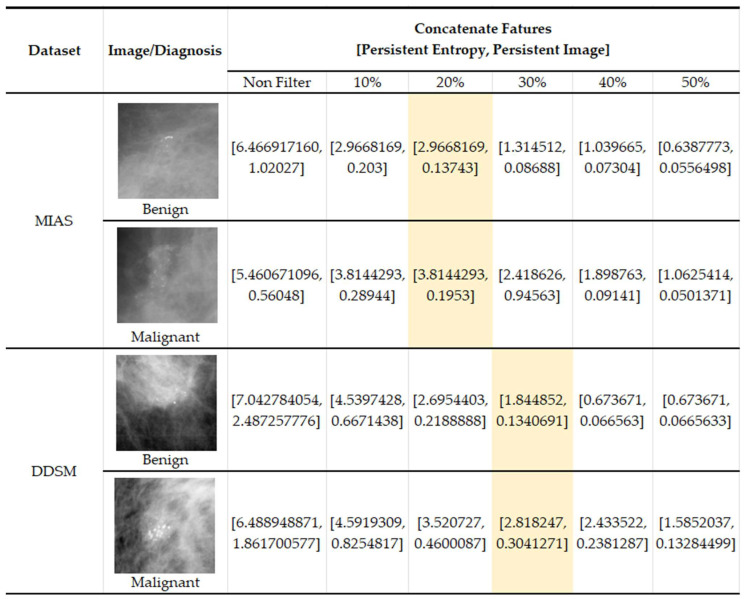
Examples of images with topological feature values in the MIAS [7] and DDSM datasets [32].

In Figure 13, we present some examples of the images from both datasets where benign images have higher values (PI and PE) in non-filter topological features than malignant features. This results in misclassification between the two classes and impacts the classification performance. Because benign microcalcifications present a long lifespan in the PD (refer to Figure 5), the significant topological features can be distinguished after filtering in the PD.

PH offers some unique advantages. One of the advantages is its ability to analyse data at multiple scales, which can be particularly useful for medical images with features of different sizes and scales. By analysing the data at multiple scales, PH can capture information about features that other methods may miss. Besides, it represents a lower computational burden to the system because the classification operation is not on the image matrix but on a compact vector from the input data [23]. This study uses two topological features for each image: PE and PI features. The complexity of a CAD system increases rapidly with the number of features used [44]. Furthermore, the proposed filtering procedure does not influence the image quality, because the process only operates on the PD, as opposed to some preprocessing methods, such as linear filtering, which can cause degradation of edges and image details, giving the images a blurred effect [13].

### Comparative Analysis

Several existing state-of-the-art non-PH models used to conduct experiments with the same MIAS and DDSM datasets were chosen for performance comparison, as shown in Table 7. Based on this table, two types of images were applied for both datasets: greyscale and binary images. Greyscale indicates that the classification process is performed straight from the original image from the dataset, consisting of 256 pixel values. Meanwhile, the classification of binary images indicates that the original images undergo a segmentation process to produce a black-and-white image, where pixel values of 0 (black) are considered as the background region and pixel values of 1 (white) are considered as segmented regions of microcalcifications. However, some microcalcifications, usually malignant cases, are not clearly visible and are closely connected to background tissue, making it impossible for segmentation algorithms to obtain complete segmentation for these calcifications [45]. For that reason, the proposed methods are tested for the greyscale images so that the whole topological structure of the image can be considered.

Various features have been studied in the literature to classify benign and malignant microcalcifications. Research by Fadil et al. [15] used 2D discrete wavelet transform for contrast enhancement of the microcalcifications and extracted eight textural features on the GLCM, achieving 95% accuracy and 0.92 AUC using the random forest (RF) classifier. On the other hand, Suhail et al. [46] present a way to obtain a single feature value by applying a scalable linear fisher discriminant analysis (LDA) approach, achieving up to 96% accuracy with 0.95 AUC using SVM. Mahmood et al. [16] employed machine learning integrated with the radiomic approach to classifying the textural and statistical features, attaining 98% accuracy and 0.90 AUC. Apart from that, Gowri et al. [17] also used textural features with fractal analysis and obtained 96.3% accuracy. Melekoodappattu et al. [5] proposed a hybrid extreme machine learning classifier consisting of the extreme learning machine (ELM) with the fruitfly optimization algorithm (ELM-FOA) along with glowworm swarm optimization (GSO). The preprocessing stage was conducted using the Wiener filter and enhanced using contrast-limited adaptive histogram equalisation (CLAHE). Here, 44 features are extracted using the speed up robust feature (SURF), Gabor filter, and GLCM, and achieved 99.15% accuracy.

In addition, topological features have also been studied by [47,48] for modelling and classification of microcalcifications, with promising results. To the best of our knowledge, our proposed method is the first work using a persistent diagram as a filtering approach as well as PH features to tackle the challenge of discriminating between benign and malignant microcalcifications. This method achieves 96.2% accuracy with 0.96 AUC for the MIAS dataset and 99.3% accuracy with 0.99 AUC for the DDSM dataset. This is comparable to other state-of-the-art non-PH approaches developed to solve the same problem.

Although the performance is satisfactory, this study has several limitations. First, this study used a limited number of images. Hence, additional testing must be conducted on mammographic images collected from hospitals or population-screening projects. Increasing the number of images would permit a more in-depth assessment and can prevent bias in the data. Second, external validation of model performance was not conducted. Doing so could have further demonstrated its generalisability. Third, the performance of the model is not compared to deep learning approaches owing to the small amount of data. For future studies, the persistent homology features can be extended to deep learning architectures and potentially achieve even better performance and robustness of image classification algorithms, particularly in the context of medical imaging, where complex structures and patterns are often present. Lastly, the choice of preprocessing steps, such as image enhancement or denoising, can also affect the resulting persistent homology features and introduce bias into the model. It is important to carefully consider the appropriate preprocessing steps for the specific dataset and to evaluate the impact of these steps on model performance.

## 5. Conclusions

In conclusion, this study presents an approach to classifying microcalcifications in mammogram images using PH and machine learning models. This study demonstrates that machine learning models successfully classify microcalcifications using appropriate PH filtering levels and features. In addition, filtering PD with concatenate features improves the classification accuracy of microcalcifications.

Integrating PH-based machine learning into clinical practice for breast cancer diagnosis can offer several potential benefits for patient care. The topological features extracted by PH can provide additional information that may not be captured by traditional image analysis methods, leading to a more accurate diagnosis. This, in turn, may facilitate early detection of breast cancer, ultimately reducing the number of unnecessary biopsies for patients. Additionally, accurate machine learning models can enable faster and more effective analysis of mammogram images, easing the workload of radiologists and other healthcare providers.

## Figures and Tables

**Table 1:** Classification performance of the PI feature in the MIAS dataset. CA = accuracy and Std = standard deviation.

Pers. Image Condition	NN		KNN		SVM		DT	
	CA ± Std	AUC	CA ± Std	AUC	CA ± Std	AUC	CA ± Std	AUC
Non-Filter	76.9 ± 8.3	0.76	73.1 ± 8.7	0.73	69.2 ± 9.1	0.85	73.1 ± 8.7	0.74
Filter 10%	80.8 ± 7.7	0.91	73.1 ± 8.7	0.73	73.1 ± 8.7	0.86	73.1 ± 8.7	0.74
Filter 20%	92.3 ± 5.2	0.93	88.5 ± 6.3	0.88	76.9 ± 8.3	0.93	92.3 ± 5.2	0.9
Filter 30%	76.9 ± 8.3	0.89	76.9 ± 8.3	0.77	73.1 ± 8.7	0.89	73.1 ± 8.7	0.74
Filter 40%	73.1 ± 8.7	0.72	73.1 ± 8.7	0.73	73.1 ± 8.7	0.9	76.9 ± 8.3	0.73
Filter 50%	69.2 ± 9.1	0.69	69.2 ± 9.1	0.69	73.1 ± 8.7	0.85	73.1 ± 8.7	0.68

**Table 2:** Classification performance of the PI feature in the DDSM dataset.

Pers. Image Condition	NN		KNN		SVM		DT	
	CA ± Std	AUC	CA ± Std	AUC	CA ± Std	AUC	CA ± Std	AUC
Non-Filter	71.4 ± 3.8	0.74	50 ± 4.2	0.5	59.3 ± 4.2	0.69	63.6 ± 4.1	0.68
Filter 10%	86.4 ± 2.9	0.93	82.9 ± 3.2	0.83	82.9 ± 3.2	0.83	85.7 ± 3	0.92
Filter 20%	90.7 ± 2.5	0.95	86.4 ± 2.9	0.86	92.9 ± 2.2	0.94	90 ± 2.5	0.93
Filter 30%	93.6 ± 2.1	0.94	93.6 ± 2.1	0.94	93.6 ± 2.1	0.94	94.3 ± 1.9	0.95
Filter 40%	91.4 ± 2.4	0.97	87.1 ± 2.8	0.87	89.3 ± 2.6	0.97	87.1 ± 2.8	0.85
Filter 50%	81.4 ± 3.3	0.91	85 ± 3	0.85	82.9 ± 3.2	0.93	83.6 ± 3.1	0.88

**Table 3:** Classification performance of the PE feature in the MIAS dataset.

Pers. Entropy Condition	NN		KNN		SVM		DT	
	CA ± Std	AUC	CA ± Std	AUC	CA ± Std	AUC	CA ± Std	AUC
Non-Filter	53.8 ± 9.8	0.55	50 ± 9.8	0.5	69.2 ± 9.1	0.78	46.2 ± 9.8	0.59
Filter 10%	57.7 ± 9.7	0.7	65.4 ± 9.3	0.65	65.4 ± 9.3	0.79	65.4 ± 9.3	0.69
Filter 20%	84.6 ± 7.1	0.9	92.3 ± 5.2	0.92	80.8 ± 7.7	0.89	73.1 ± 8.7	0.75
Filter 30%	80.8 ± 7.7	0.83	80.8 ± 7.7	0.81	80.8 ± 7.7	0.89	80.8 ± 7.7	0.8
Filter 40%	76.9 ± 8.3	0.81	80.8 ± 7.7	0.81	76.9 ± 8.3	0.92	84.6 ± 7.1	0.79
Filter 50%	76.9 ± 8.3	0.79	73.1 ± 8.7	0.73	80.8 ± 7.7	0.89	88.5 ± 6.3	0.83

**Table 4:** Classification performance of the PE feature in the DDSM dataset.

Pers. Entropy Condition	NN		KNN		SVM		Decision Tree	
	CA ± Std	AUC	CA ± Std	AUC	CA ± Std	AUC	CA ± Std	AUC
Non-Filter	50.7 ± 4.2	0.55	50 ± 4.2	0.5	49.3 ± 4.2	0.53	55.7 ± 4.2	0.54
Filter 10%	87.9 ± 2.8	0.92	79.3 ± 3.4	0.79	87.9 ± 2.8	0.94	80.7 ± 3.3	0.84
Filter 20%	92.1 ± 2.3	0.96	91.4 ± 2.4	0.94	93.6 ± 2.1	0.97	90.7 ± 2.5	0.95
Filter 30%	95 ± 1.8	0.97	96.4 ± 1.6	0.98	95.7 ± 1.7	0.99	92.9 ± 2.2	0.94
Filter 40%	92.1 ± 2.3	0.97	89.3 ± 2.6	0.89	94.3 ± 1.9	0.97	90.7 ± 2.5	0.89
Filter 50%	85.7 ± 2.9	0.93	82.9 ± 3.2	0.83	87.9 ± 2.8	0.97	85.7 ± 3	0.91

**Table 5:** Classification performance of concatenating features in the MIAS dataset.

Concatenate Features	NN		KNN		SVM		DT	
	CA ± Std	AUC	CA ± Std	AUC	CA ± Std	AUC	CA ± Std	AUC
No Filter	61.5 ± 9.5	0.6	53.8 ± 9.8	0.54	69.2 ± 9.1	0.82	69.2 ± 9.1	0.68
Filter 10%	61.5 ± 9.5	0.67	53.8 ± 9.8	0.54	69.2 ± 9.1	0.79	73.1 ± 8.7	0.74
Filter 20%	96.2 ± 3.7	0.96	92.3 ± 5.2	0.92	88.5 ± 6.3	0.93	92.3 ± 5.2	0.91
Filter 30%	84.6 ± 7.1	0.84	80.8 ± 7.7	0.81	80.8 ± 7.7	0.89	84.6 ± 7.1	0.82
Filter 40%	84.6 ± 7.1	0.83	80.8 ± 7.7	0.81	80.8 ± 7.7	0.89	84.6 ± 7.1	0.77
Filter 50%	76.9 ± 8.3	0.81	80.8 ± 7.7	0.81	80.8 ± 7.7	0.89	84.6 ± 7.1	0.8

**Table 6:** Classification performance of concatenating features in the DDSM dataset.

Concatenate Features	NN		KNN		SVM		DT	
	CA ± Std	AUC	CA ± Std	AUC	CA ± Std	AUC	CA ± Std	AUC
No Filter	71.4 ± 3.8	0.75	73.6 ± 3.7	0.79	75 ± 3.7	0.81	68.6 ± 3.9	0.73
Filter 10%	90 ± 2.5	0.95	90 ± 2.5	0.9	91.4 ± 2.4	0.96	90 ± 2.5	0.91
Filter 20%	94.3 ± 1.9	0.95	94.3 ± 1.9	0.94	97.1 ± 1.4	0.99	92.9 ± 2.2	0.93
Filter 30%	97.9 ± 1.2	0.98	99.3 ± 0.7	0.99	98.6 ± 1	0.99	97.9 ± 1.2	0.98
Filter 40%	92.9 ± 2.2	0.95	92.1 ± 2.3	0.91	95 ± 1.8	0.99	96.4 ± 1.6	0.95
Filter 50%	91.4 ± 2.4	0.94	87.9 ± 2.8	0.88	87.9 ± 2.8	0.97	87.9 ± 2.7	0.9

**Table 7:** Comparative analysis with other methods.

Method	Features	Image Type	Dataset	Classifier	Result
Fadil et al. [15]	Texture (GLCM)	Greyscale	DDSM	DWT-RF	CA = 95%, AUC = 0.92
Suhail et al. [46]	Local Features	Binary	DDSM	LDA-SVM	CA = 96%, AUC = 0.95
Mahmood et al. [16]	Textural and Statistical	Binary	MIAS	Radiomic-SVM	CA = 98%, AUC = 0.90
Gowri et al. [17]	Textural with Fractal Analysis	Binary	MIAS	NN	CA = 96.3%, AUC = NA
Melekoodappattu et al. [5]	SURF, Gabor, and GLCM	Greyscale	MIAS	GSO-ELM-FOA	CA = 99.15%, AUC = NA
Chen et al. [47]	Multiscale Morphology Graph	Binary	MIAS	KNN	CA = 95%, AUC = 0.96
			DDSM	KNN	CA = 85.2%, AUC = 0.90
Strange et al. [48]	Mereotopological Barcode	Binary	MIAS	KNN	CA = 95%, AUC = 0.96
			DDSM	KNN	CA = 80%, AUC = 0.82
Proposed Approach	PI and PE Features	Greyscale	MIAS	NN	CA = 96.2%, AUC = 0.96
			DDSM	KNN	CA = 99.3%, AUC = 0.99

## Data Availability

The public datasets are available in the publicly accessible repository; refer to [7] for the MIAS and [32] DDSM datasets.

## References

[1] Ferlay J., Colombet M., Soerjomataram I., Parkin D.M., Piñeros M., Znaor A., Bray F. (2021). Cancer Statistics for the Year 2020: An Overview. Int. J. Cancer.

[2] Vy V.P.T., Yao M.M.-S., Le N.Q.K., Chan W.P. (2022). Machine Learning Algorithm for Distinguishing Ductal Carcinoma in Situ from Invasive Breast Cancer. Cancers.

[3] Ramadan S.Z. (2020). Methods Used in Computer-Aided Diagnosis for Breast Cancer Detection Using Mammograms: A Review. J. Healthc. Eng..

[4] Htay M.N.N., Donnelly M., Schliemann D., Loh S.Y., Dahlui M., Somasundaram S., Tamin N.S.B.I., Su T.T. (2021). Breast Cancer Screening in Malaysia: A Policy Review. Asian Pac. J. Cancer Prev..

[5] Melekoodappattu J.G., Subbian P.S., Queen M.P.F. (2021). Detection and Classification of Breast Cancer from Digital Mammograms Using Hybrid Extreme Learning Machine Classifier. Int. J. Imaging Syst. Technol..

[6] Oliver A., Torrent A., Lladó X., Tortajada M., Tortajada L., Sentís M., Freixenet J., Zwiggelaar R. (2012). Automatic Microcalcification and Cluster Detection for Digital and Digitised Mammograms. Knowl. Based Syst..

[7] Suckling J. (1994). The Mammographic Image Analysis Society Digital Mammogram Database. Exerpta Med. Int. Congr..

[8] Azam A.S.B., Malek A.A., Ramlee A.S., Suhaimi N.D.S.M., Mohamed N. Segmentation of Breast Microcalcification Using Hybrid Method of Canny Algorithm with Otsu Thresholding and 2D Wavelet Transform. Proceedings of the 2020 10th IEEE International Conference on Control System, Computing and Engineering (ICCSCE).

[9] Dabass J., Arora S., Vig R., Hanmandlu M. Segmentation Techniques for Breast Cancer Imaging Modalities-A Review. Proceedings of the 2019 9th International Conference on Cloud Computing, Data Science & Engineering (Confluence).

[10] Banumathy D., Khalaf O.I., Romero C.A.T., Raja P.V., Sharma D.K. (2023). Breast Calcifications and Histopathological Analysis on Tumour Detection by CNN. Comput. Syst. Sci. Eng..

[11] Roty S., Wiratkapun C., Tanawongsuwan R., Phongsuphap S. Analysis of Microcalcification Features for Pathological Classification of Mammograms. Proceedings of the 2017 10th Biomedical Engineering International Conference (BMEiCON).

[12] Fan L., Zhang F., Fan H., Zhang C. (2019). Brief Review of Image Denoising Techniques. Vis. Comput. Ind. Biomed. Art.

[13] Krishnan A. (2016). An Overview of Mammogram Noise and Denoising Techniques. Int. J. Eng. Res. Gen. Sci..

[14] Patil R.S., Biradar N. (2020). Automated Mammogram Breast Cancer Detection Using the Optimized Combination of Convolutional and Recurrent Neural Network. Evol. Intell..

[15] Fadil R., Jackson A., El Majd B.A., El Ghazi H., Kaabouch N. Classification of Microcalcifications in Mammograms Using 2D Discrete Wavelet Transform and Random Forest. Proceedings of the 2020 IEEE International Conference on Electro Information Technology (EIT).

[16] Mahmood T., Li J., Pei Y., Akhtar F., Imran A., Yaqub M. (2021). An Automatic Detection and Localization of Mammographic Microcalcifications ROI with Multi-Scale Features Using the Radiomics Analysis Approach. Cancers.

[17] Gowri V., Valluvan K.R., Vijaya Chamundeeswari V. (2018). Automated Detection and Classification of Microcalcification Clusters with Enhanced Preprocessing and Fractal Analysis. Asian Pac. J. Cancer Prev..

[18] Pun C.S., Lee S.X., Xia K. (2022). Persistent-Homology-Based Machine Learning: A Survey and a Comparative Study. Artif. Intell. Rev..

[19] Choe S., Ramanna S. (2022). Cubical Homology-Based Machine Learning: An Application in Image Classification. Axioms.

[20] Asaad A., Ali D., Majeed T., Rashid R. (2022). Persistent Homology for Breast Tumor Classification Using Mammogram Scans. Mathematics.

[21] Kusano G., Fukumizu K., Hiraoka Y. (2018). Kernel Method for Persistence Diagrams via Kernel Embedding and Weight Factor. J. Mach. Learn. Res..

[22] Moroni D., Pascali M.A. (2021). Learning Topology: Bridging Computational Topology and Machine Learning. Pattern Recognit. Image Anal..

[23] Avilés-Rodríguez G.J., Nieto-Hipólito J.I., Cosío-León M.D.L.Á., Romo-Cárdenas G.S., Sánchez-López J.D.D., Radilla-Chávez P., Vázquez-Briseño M. (2021). Topological Data Analysis for Eye Fundus Image Quality Assessment. Diagnostics.

[24] Adams H., Emerson T., Kirby M., Neville R., Peterson C., Shipman P., Chepushtanova S., Hanson E., Motta F., Ziegelmeier L. (2017). Persistence Images: A Stable Vector Representation of Persistent Homology. J. Mach. Learn. Res..

[25] Teramoto T., Shinohara T., Takiyama A. (2020). Computer-Aided Classification of Hepatocellular Ballooning in Liver Biopsies from Patients with NASH Using Persistent Homology. Comput. Methods Programs Biomed..

[26] Oyama A., Hiraoka Y., Obayashi I., Saikawa Y., Furui S., Shiraishi K., Kumagai S., Hayashi T., Kotoku J. (2019). Hepatic Tumor Classification Using Texture and Topology Analysis of Non-Contrast-Enhanced Three-Dimensional T1-Weighted MR Images with a Radiomics Approach. Sci. Rep..

[27] Leykam D., Rondón I., Angelakis D.G. (2022). Dark Soliton Detection Using Persistent Homology. Chaos Interdiscip. J. Nonlinear Sci..

[28] Edwards P., Skruber K., Milićević N., Heidings J.B., Read T.A., Bubenik P., Vitriol E.A. (2021). TDAExplore: Quantitative Analysis of Fluorescence Microscopy Images through Topology-Based Machine Learning. Patterns.

[29] Nishio M., Nishio M., Jimbo N., Nakane K. (2021). Homology-Based Image Processing for Automatic Classification of Histopathological Images of Lung Tissue. Cancers.

[30] Rammal A., Assaf R., Goupil A., Kacim M., Vrabie V. (2022). Machine Learning Techniques on Homological Persistence Features for Prostate Cancer Diagnosis. BMC Bioinform..

[31] Conti F., Moroni D., Pascali M.A. (2022). A Topological Machine Learning Pipeline for Classification. Mathematics.

[32] Heath M., Bowyer K., Kopans D., Moore R., Kegelmeyer P. The Digital Database for Screening Mammography. Proceedings of the Fifth International Workshop on Digital Mammography.

[33] Beksi W.J., Papanikolopoulos N. 3D Region Segmentation Using Topological Persistence. Proceedings of the 2016 IEEE/RSJ International Conference on Intelligent Robots and Systems (IROS).

[34] Otter N., Porter M.A., Tillmann U., Grindrod P., Harrington H.A. (2017). A Roadmap for the Computation of Persistent Homology. EPJ Data Sci..

[35] Kramár M., Levanger R., Tithof J., Suri B., Xu M., Paul M., Schatz M.F., Mischaikow K. (2016). Analysis of Kolmogorov Flow and Rayleigh–Bénard Convection Using Persistent Homology. Physica D.

[36] Garin A., Tauzin G. A Topological “reading” Lesson: Classification of MNIST Using TDA. Proceedings of the 2019 18th IEEE International Conference on Machine Learning and Applications, ICMLA 2019.

[37] Pun C.S., Xia K., Lee S.X. (2018). Persistent-Homology-Based Machine Learning and Its Applications—A Survey. arXiv.

[38] Chazal F., Michel B. (2021). An Introduction to Topological Data Analysis: Fundamental and Practical Aspects for Data Scientists. Front. Artif. Intell..

[39] Atienza N., Gonzalez-Díaz R., Soriano-Trigueros M. (2020). On the Stability of Persistent Entropy and New Summary Functions for Topological Data Analysis. Pattern Recognit..

[40] Moon C., Li Q., Xiao G. (2020). Using Persistent Homology Topological Features to Characterize Medical Images: Case Studies on Lung and Brain Cancers. arXiv.

[41] Jiao Y., Du P. (2016). Performance Measures in Evaluating Machine Learning Based Bioinformatics Predictors for Classifications. Quant. Biol..

[42] Kaji S., Sudo T., Ahara K. (2020). Cubical Ripser: Software for Computing Persistent Homology of Image and Volume Data. arXiv.

[43] Turkes R., Nys J., Verdonck T., Latre S. (2021). Noise Robustness of Persistent Homology on Greyscale Images, across Filtrations and Signatures. PLoS ONE.

[44] Sakka E., Prentza A., Koutsouris D. (2006). Classification Algorithms for Microcalcifications in Mammograms (Review). Oncol. Rep..

[45] Temmermans F., Jansen B., Willekens I., Van de Casteele E., Deklerck R., Schelkens P., De Mey J., Tescher A.G. (2013). Classification of Microcalcifications Using Micro-CT. Proceedings of the Applications of Digital Image Processing XXXVI.

[46] Suhail Z., Denton E.R.E., Zwiggelaar R. (2018). Classification of Micro-Calcification in Mammograms Using Scalable Linear Fisher Discriminant Analysis. Med. Biol. Eng. Comput..

[47] Chen Z., Strange H., Oliver A., Denton E.R.E., Boggis C., Zwiggelaar R. (2015). Topological Modeling and Classification of Mammographic Microcalcification Clusters. IEEE Trans. Biomed. Eng..

[48] Strange H., Chen Z., Denton E.R.E., Zwiggelaar R. (2014). Modelling Mammographic Microcalcification Clusters Using Persistent Mereotopology. Pattern Recognit. Lett..

